# Particle Filter Tracking System Based on Digital Zoom and Regional Image Measure

**DOI:** 10.3390/s25030880

**Published:** 2025-01-31

**Authors:** Qisen Zhao, Liquan Dong, Xuhong Chu, Ming Liu, Lingqin Kong, Yuejin Zhao

**Affiliations:** 1 Beijing Key Laboratory for Precision Optoelectronic Measurement Instrument and Technology, School of Optics and Photonics, Beijing Institute of Technology, Beijing 100081, Chinachuxuhong001@bit.edu.cn (X.C.);; 2 Yangtze Delta Region Academy of Beijing Institute of Technology, Jiaxing 314019, China

**Keywords:** object tracking, particle filter, regional image measure, digital zoom

## Abstract

To address the challenges of low accuracy and the difficulty in balancing a large field of view and long distance when tracking high-speed moving targets with a single sensor, an ROI adaptive digital zoom tracking method is proposed. In this paper, we discuss the impact of ROI on image processing and describe the design of the ROI adaptive digital zoom tracking system. Additionally, we construct an adaptive ROI update model based on normalized target information. To capture target changes effectively, we introduce the multi-scale regional measure and propose an improved particle filter algorithm, referred to as the improved multi-scale regional measure resampling particle filter (IMR-PF). This method enables high temporal resolution processing efficiency within a high-resolution large field of view, which is particularly beneficial for high-resolution videos. The IMR-PF can maintain high temporal resolution within a wide field of view with high resolution. Simulation results demonstrate that the improved target tracking method effectively improves tracking robustness to target motion changes and reduces the tracking center error by 20%, as compared to other state-of-the-art methods. The IMR-PF still maintains good performance even when confronted with various interference factors and in real-world scenario applications.

## 1. Introduction

Single object detection and tracking (SODT) technology has important applications in video surveillance, target analysis, smart ice and snow sports, and other fields. The technology has garnered widespread attention from scholars and industries worldwide. However, achieving a balance between wide viewing angles and high resolution in machine vision has always been a challenge. Maintaining spatial resolution and temporal resolution over long distances remains a key hurdle in the field of machine vision. Capturing, processing, and transmitting images with higher spatial resolution requires more time, consequently reducing temporal resolution [1]. The accuracy of an algorithm can be significantly influenced by spatial resolution [2], with even minor variations in it leading to notable consequences [3]. Both high temporal and spatial resolutions are highly desirable for machine vision [4], as they can enhance the precision of object detection and tracking [5].

The current single-target tracking methods mainly comprise discriminant correlation filtering methods [6] and methods based on deep learning [7]. Discriminant correlation filtering methods typically adopt the framework of Kalman filtering [8], which demonstrates superior tracking performance for linear and Gaussian motion. However, most real-world motion is nonlinear. The particle filter (PF) is employed for nonlinear motion filtering and prediction, as it is unaffected by linear and Gaussian distribution [9]. Nevertheless, the time complexity and particle degradation associated with PF can easily result in filtering processes falling into local optima, thus hindering the tracking accuracy of nonlinear motion and non-Gaussian targets [10]. Some researchers have introduced swarm intelligence to particle filtering to address the issue of particles getting stuck in local optima. The firefly particle filter algorithm reduces the time complexity and local optimum phenomenon by combining the firefly algorithm and the particle filter algorithm [11]. Furthermore, deep learning-based methods improve tracking accuracy by matching target features using a data-driven approach [12]. However, when applied to high-resolution images, these methods often sacrifice temporal resolution.

The experimental verification and datasets used in these methods are typically limited to small size changes in videos or images and cannot be readily applied to tracking scenarios with a wide dynamic range of distance and resolution.

A study achieves the region of interest (ROI) tracking within a large field of view by setting ROI [13]. However, this method is limited to targets with small motion changes at close range and is not suitable for tracking and shooting high-speed moving targets in ice and snow sports.

In this paper, we propose a particle filter tracking system based on digital zoom and regional image measure. Our contributions are as follows:

(1) We introduce a technique for adaptive digital zoom that dynamically adjusts the location and size of the region of interest (ROI), based on the target’s nonlinear normalized center distance and aspect ratio. This enables real-time detection and tracking within a broad field of view and high dynamic range. The spatial and temporal resolution of the target tracking process is enhanced.
(2) In addition, we introduce a particle filter tracking algorithm that integrates multi-scale regional measures into the resampling process. By constructing a multi-scale regional measure feature module, the resampling is improved according to the target change state, and the extended Kalman filter (EKF) is applied to improve the importance density of the particle filter. The improved method removes the interference of motion mutations and improves the stability and accuracy of target tracking.
(3) Furthermore, our method achieves impressive results on public benchmark test sets. It outperforms existing methods, including deep feature matching and trackers based on correlation filters, in terms of target tracking accuracy and tracking error. Our method also attains state-of-the-art performance for object tracking tasks in scenes with complex motion variations and large distance variations, thereby demonstrating the general applicability of our approach.

The rest of this paper is organized as follows: Section 2 gives an overview of related work; Section 3 explains in detail the proposed particle filter tracking system based on digital zoom and regional image measure; Section 4 describes the experimental setup and presents the results; and finally, Section 5 concludes this paper.

## 2. Related Works

### 2.1. Application of ROI

The utilization of ROI is widely employed in various detection and tracking applications as a means of reducing noise interference in non-target areas. In the field of medical imaging, there are technologies such as remote photoplethysmography [14], detection of hand veins and handprints [15], along with blink monitoring for patients with ALS [16], all of which need to be performed only in high-quality diagnostic ROI areas [17]. For computing-intensive fields, aerial surveillance transmission of ROI images can substantially decrease the data volume collected by drones [18], and information reconstruction on the ROI can also greatly speed up processing [19]. Lane detection applications also benefit from processing smaller ROIs instead of the entire image, enabling real-time detection, which is particularly important for transportation warning systems [20]. Correlation filtering-based target tracking methods also adopt ROI technology. The object of required is detected and captured, and an estimation filter is utilized to employ the ROI position in the subsequent frame. However, fixed ROIs often limit the applicability of these methods, and dynamic signal ranges often necessitate adaptively changing ROIs. The selection strategy for ROIs has also been a topic of discussion among researchers. Manual selection requires a stable signal state. Certain studies have employed partition coding on images, hidden Markov chains, and Gaussian mixture models to differentiate between the ROI and background unrelated to road traffic [21]. Kiadtikorn and Tatnall [22] and Ma et al. [23] separately implemented the histogram segmentation technique and a deep learning model enhanced with attention mechanisms to identify regions of interest (ROIs) within remote sensing data. However, the large field of view and high resolution make ROI extraction a more complex task. Therefore, we propose an ROI selection method that is adaptive to changes in the field of view, accomplished through the normalization of the target distance and aspect ratio of the detection results.

### 2.2. Target Tracking

Target tracking faces several challenges, including occlusion (OCC), deformation (DEF), camera motion (BC), illumination change (IV), and rapid motion (MB). Numerous methods have been proposed to address these problems.

For the tasks of predicting trajectories of high-speed maneuvering targets, filtering algorithms such as Kalman filtering (KF) are commonly utilized to process original information like the relative distance and angle of the target [24]. These algorithms are then combined with target motion models to enable accurate tracking of target trajectories. The presence of nonlinearity in the system can lead to a loss of accuracy, particularly in cases of high nonlinearity where the estimation results of the extended Kalman filter (EKF) may exhibit significant errors [25]. To mitigate these challenges, Sort introduced a method that integrates Kalman filtering with the Hungarian algorithm to enhance target motion estimation and data association accuracy [26]. Additionally, the ByteTrack algorithm was developed to account for both low-confidence and high-confidence detection boxes, employing different strategies based on the similarity of detection boxes to enhance tracking performance [27].

The advancement of deep learning models has facilitated the formulation of the Siam RPN++ algorithm, which leverages twin networks to match deep features of the target, thereby improving the accuracy of target tracking [12]. Furthermore, researchers had also improved the accuracy of target tracking by introducing feature combination modules [28] and different attention mechanisms [29]. Sumaira et al. introduced the SPT model [30], which incorporates a re-identification module specifically designed for pedestrian targets. By integrating metric learning, they enhanced the tracking performance of the Siamese model. However, the model’s reliance on shared weights for extracting similar features makes it vulnerable to distortions caused by scene variations. Addressing this limitation, Feng et al. developed a solution by integrating multiple attention mechanisms with background features [31], enabling the model to adaptively update and better handle diverse target changes in complex environments. The Transformer-based single object tracking (SOT) model stands out for its straightforward architecture and optimal balance between performance and speed. To tackle the issue of targets moving out of the field of view, Miao et al. implemented a backtracking recognition module alongside a trajectory disappearance discrimination model [32], also taking into account fine-grained low-level target features. Further advancing the field, Liang et al. proposed the Multi Local Guided Tracker (MLGT) [33], which enhances target modeling and representation through the fusion of multi-level and multi-stream features. Notably, to ensure real-time processing, the model optimizes computational efficiency by reducing the size of processed images and search areas.

Despite these advancements, the effectiveness of these methods is often compromised by several challenges, including limited search areas, the absence of template updates, and sample imbalance. These factors render the models susceptible to variations in the field of view and the deformation of small-sized targets.

Particle filtering (PF) leverages random samples to estimate the probability density function, and the integral operation is replaced by the sample average to obtain the target state with the minimum variance estimate [34]. PF is particularly effective in handling nonlinear and non-Gaussian target tracking challenges due to the randomness of particles. To address issues like particle weight degradation, researchers have explored the integration of swarm intelligence, including particle swarm optimization (PSO) [35] and firefly algorithms [36], for locating high-likelihood areas.

However, this type of optimization method may be attracted by local optimal values during the iterative search process, and the global search ability will be reduced. The kernel-correlated particle filter [37] technique incorporates the Histogram of Oriented Gradients (HOG) [38] feature for enhanced discrimination and has demonstrated effectiveness in tracking partially occluded and rotated targets, underscoring the importance of image features in target tracking.

When applied to scenarios with large fields of view, deep learning models often face limitations due to their low resolution, particularly when tracking small targets that undergo significant changes. These target variations further constrain the robustness and accuracy of traditional particle filtering improvement methods. To address these challenges, we propose an enhanced particle filtering algorithm that incorporates image change features measured within the target area. By leveraging a Kalman filter to approximate the particle importance density function, we construct a robust and high-precision tracking framework. This approach, when integrated with an adaptive variable region of interest (ROI) module, enables real-time processing of high-resolution images from large scenes while maintaining the robustness and accuracy of target tracking. The proposed method effectively balances computational efficiency and tracking performance, making it well-suited for applications requiring precise tracking in complex and dynamic environments.

## 3. Methodology

### 3.1. Adaptive Digital Zoom Tracking System

This study aims to address the challenge of accurate detection and tracking of targets with wide-ranging changes of distance and size changes. For instance, in scenarios like tracking skiers in large outdoor venues, it is crucial to take into account both small targets at long distances and large targets at close ranges. To address this issue, we propose a particle filter tracking method based on digital zoom and image area measurement, which forms the core of a novel object detection and tracking system capable of tracking targets of varying sizes and distances within a large field of view.

**Figure 1:**
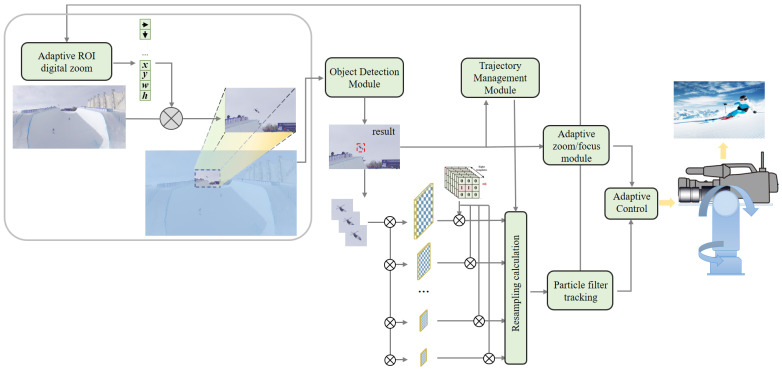
The overall structure of the proposed method.

The overall workflow of the proposed method is illustrated in Figure 1. The system primarily consists of two key modules. First, the region of interest (ROI) Adaptive Digital Zoom module adjusts the ROI in the input image based on the detection and tracking results. This refined region is then fed into a universal object detection module to obtain target information. Second, the target details (including size and position) generated by the object detection module are passed to the object tracking module, which employs an improved multi-scale regional measure resampling particle filter (IMR-PF) to address potential target omission issues. To implement this method, we developed a hardware system, as shown on the right side of Figure 1. The system comprises a high-resolution camera and a pan-tilt (P/T) rotary turntable. The high-resolution camera captures wide field-of-view images, which are processed for adaptive ROI digital zoom, object detection, and variable particle filter tracking.

The rotating turntable is designed to synchronize with the movement of small targets, ensuring that it consistently points toward them. It serves as a platform for high-resolution cameras and other narrow field-of-view sensors, enabling the capture of higher-quality images and facilitating close-up imaging of small targets. To achieve this functionality, the target’s center position, calculated by the IMR-PF method, is converted into control signals. These signals drive the turntable, enabling real-time pan-tilt (P/T) rotation and precise target tracking. Additionally, the detection results generated during the target information calculation process are stored in the trajectory management submodule for further analysis and utilization.

To validate the effectiveness of the algorithm in practical applications, we selected a large skiing venue with a range of 0–170 m as one of the test scenarios. Based on the single-aperture imaging model, we chose a high-resolution, high-frame-rate, large field-of-view industrial camera (MS-XG903C/M camera produced by MINDVISION in Shenzhen, China) as the visual sensor. This setup ensures robust performance in capturing and tracking targets across varying distances and sizes in a dynamic environment.

(1) $$h_{i}=\frac{f}{d}h_{o}$$

where f is the focal length of the visual sensor, d is the object distance, $h_{o}$ is the image height, and $h_{i}$ is the object height. Its resolution is 4208 × 2160, the field of view is 40.2 × 30.6, the focal length is 8 mm, and the imaging frame rate is 100 fps. We selected a 45× zoom variable focal length camera (The Sony PXW-Z750 camera produced by Sony in Tokyo, Japan) for close-up shooting.

### 3.2. ROI Adaptive Digital Zoom Algorithm

In a scene with a large field of view, the size of the detection resolution can impact the detection speed. Whether the ROI is either too large or too small, the detection of the target becomes challenging. It is crucial to choose a suitable ROI. The ROI adaptive digital zoom algorithm processes the original picture of the visual sensor, and it updates the ROI in real time based on the detection and tracking results. The updated ROI is then used to extract the image in the picture with original resolution. The extracted image is digitally zoomed for target detection.

In the adaptive ROI algorithm, the image of the entire field of view (FOV) captured by the visual sensor is divided into m × n partitions, and each partition is subjected to cyclic detection using the detection algorithm (ROI ≤ FOV). When a target is detected, the algorithm generates an ROI based on the detection results and predicts the ROI for the subsequent frame using both detection and tracking results. In this method, m = 6 and n = 4 are selected for partitioning. During the process of target change, the region of interest (ROI) must be updated in advance to ensure the target is correctly included in the next frame. This pre-update is performed adaptively based on changes in target information and can be modeled as a regression relationship with the target’s characteristics.

The regression relationship has been proven to require careful consideration of factors such as the error distance and aspect ratio between the predicted target and the true target [39]. Therefore, the size-adaptive update of the region of interest (ROI) is represented by Equation (2), which ensures that the ROI adjusts proportionally to changes in the size factor (representing the width and height of the target) and the error distance factor. Specifically, when the error distance increases, the size of the ROI is expanded proportionally to ensure the target remains within the ROI area. The equation is expressed as follows:

(2) $$ROI_{new}=S\left({ROI}_{old},w,h\right)+\beta D\left(ROI_{old},x,y\right)$$

Among them, $ROI_{old}$ is the previous ROI, w,h are the width and height of the target box, x,y are the center positions of the target box, $S\left(\cdot \right)$ is the update function based on the size factor, $D\left(\cdot \right)$ is the update function based on the position factor, and the weighting factors are represented by β=0.4.

The target size not only impacts the effectiveness of target detection and tracking but also influences the subjective perception of the observer regarding the image display. In ROI updates, the size adjustment is determined by multiplying two ratios: the change in target height between consecutive frames $h^{k}/h^{k-1}$ and the ratio of the target height in the previous frame $h^{k-1}$ to the preset height, as shown in Equation (3). When the target height in the current frame exceeds that of the previous frame, the ROI size (without considering the aspect ratio and distance factors) should be increased. Additionally, the target size affects not only detection and tracking performance but also the observer’s subjective perception of the image display. To mitigate these effects, our objective is to adjust the target height to approach a preset height of 360 pixels after ROI updates. To prevent the target from overflowing the boundaries excessively, we introduced a minimum of 20% redundant space (α is the size bias factor, and α=0.2 in this method).

(3) $$ROI=\frac{h^{k}}{h^{k-1}}\times \left(\frac{h^{k-1}}{360}+\alpha \right)\times ROI_{old}$$

Inspired by the CIOU [39], updating the ROI size directly without considering aspect ratio factors can make the system susceptible to the influence of target rotation. When the target rotates, its motion dynamics change significantly, which may cause the target to rapidly move outside the ROI. To address this, we incorporated a multiplier for aspect ratio consistency into Equation (3). This multiplier expands the ROI when the target rotates, ensuring that the target remains within the detection area. Furthermore, a normalized centroid distance multiplier was added to Equation (3). When the predicted target center deviates significantly from the actual target center, the normalized centroid distance increases, prompting the ROI to expand further. This prevents the target from moving too quickly and overflowing the image boundaries. This approach enhances the stability of the algorithm. The ROI update equation (Equation (4)), which incorporates improvements based on the position factor and size factor, is as follows:

(4) $$\begin{array}{l} S=\left(\frac{4}{\pi^{2}}{\left(\arctan\frac{w^{k-1}}{h^{k-1}}-\arctan\frac{w}{h}\right)}^{2}\right)\times \frac{h^{k}}{h^{k\text{-}1}}\times \left(\frac{h^{k-1}}{360}+\alpha \right)\times ROI_{old}\\ D=\frac{\rho^{2}\left(b,b^{k-1}\right)}{c^{2}}\times \frac{h^{k}}{h^{k\text{-}1}}\times \left(\frac{h^{k-1}}{360}+\alpha \right)\times ROI_{old} \end{array}$$

Among them, b=[x,y] and $b^{k-1}=[x^{k-1},y^{k-1}]$ are the center points of the target boxes of adjacent images, c is the diagonal length of the target box, and ρ is the Euclidean distance.

During the target detection process, the target detection failure delay threshold $t_{delay}$ is set considering the case of target loss for a long time. When the target loss time exceeds $t_{delay}$, we increase the ROI to prevent further loss. In the tracking state, the ROI follows the movement of the target to ensure it remains centered within the ROI. Consequently, the positional distribution of the target within the image resembles a Gaussian distribution. We propose an ROI size adaptive update method based on time and position changes to adapt to the target loss situation, and the position change factor γ is calculated by the improved Gaussian distribution. If the target deviates significantly from the center position in the current frame, the ROI scaling amount increases. Conversely, if the target is stable, the ROI scaling amount is appropriately reduced. To ensure that the position change factor is 1 when the target position distance from the center is 0, the position change factor is calculated using Equation (5), which leverages the properties of the normalized Gaussian distribution (with a range of [0, 1]). The equation is defined as follows:

(5) $$\gamma =\frac{1}{\sqrt{2\pi }\sigma }\left(2-\exp\left(-\frac{{\left(b-b_{cen}\right)}^{2}}{2\sigma^{2}}\right)\right)$$

Among them, $b_{cen}=[x_{cen},y_{cen}]$ is the center point of the picture and σ is the target deviation variance.

Therefore, when the target loss time exceeds the threshold, the position factor is primarily updated based on the time factor and the Gaussian distribution improvement. Conversely, the ROI size update method is updated based on the aspect ratio and distance factor improvement. The general ROI size adaptive update equation (Equation (5)) is derived by simplifying and combining Equations (3) and (4), as follows:

(6) $$ROI_{new}=\left\{\begin{array}{l} \frac{t_{loss}}{t_{delay}+0.001}\times \frac{1}{\sqrt{2\pi }\sigma }\left(2-\exp\left(-\frac{{\left(x-\mu \right)}^{2}}{2\sigma^{2}}\right)\right)\times ROI_{old},t_{loss}>t_{delay}\\ \left(\frac{\rho^{2}\left(b,b^{k-1}\right)}{c^{2}}+\alpha \frac{4}{\pi^{2}}\left(\arctan\frac{w^{k-1}}{h^{k-1}}-\arctan\frac{w}{h}\right)\right)\times \frac{h^{k}}{h^{k\text{-}1}}\times \left(\frac{h^{k-1}}{360}+\alpha \right)\times ROI_{old},\;\mathrm{others} \end{array}\right.$$

In addition, we hope that the position of the ROI can also change adaptively with the detection results. At the same time, to enhance the stability of ROI position adjustments, this study employs a method that updates the ROI position through nonlinear normalization of the center point distance, leading to improved stability in target detection.

(7) $$RoI_{pos}=\left(x,y\right)+\Delta \left(x,y\right)\cdot \mathrm{sigmod}\left(\frac{\rho^{2}\left(b,b^{k-1}\right)}{c^{2}}\right)$$

The ROI adaptive digital zoom algorithm is implemented as Algorithm 1.
**Algorithm 1** The ROI adaptive digital zoom algorithm**Input:** x, y, w, h **Output:** ROI
**1:** Initialize(ROI, Result, Result_track);**2:** Result_init_(x, y, w, h) = Partition loop detect(img);**3:** ROI_init_ = Init_ROI(img, Result_init_);**4:** While True do:**5:**   Result(x, y, w, h, c) = Detect(img ⊗ ROI);**6:**   Result_track_(X, Y, w, h) = track(Result^−1^);**7:**   If t_loss_ > t_delay_ then:**8:**     ROI_new_ = Renew_lost_ROI(ROI^−1^, Result_track_^−1^);**9:**    Else then:**10:**    ROI_new_ = Renew_ROI(ROI^−1^, Result_track_^−1^);**11:**  End;**12:**   ROI_pos_ = Pos_renew_ROI(ROI_pos_^−1^, Result_track_^−1^);**13:**   Return ROI_new_, ROI_pos_;**14:**   If stop then:**15:**     Break;**16:** End;


### 3.3. Particle Filter Tracking Algorithm Based on Multi-Scale Regional Measure Resampling

To tackle the trajectory mutation issue in target tracking, this paper proposes an improved particle filter algorithm based on multi-scale regional measure resampling (IMR-PF). In IMR-PF, the target area image measure is first calculated, and then the particle filter based on the extended Kalman prediction is used. At the same time, the firefly algorithm, which leverages the image measure, is applied to assign weights to the particles, thus improving the precision of the target tracking.

#### 3.3.1. Multi-Scale Regional Feature Module

The spatial distribution of the target is continuous during the motion process, but may exhibit as discontinuous due to the motion mutation. These discontinuities can be used to identify changes in the target’s motion pattern. In the IMR-PF algorithm, the target box and its surrounding region are considered for calculating the feature distribution. Previous work [40] has demonstrated that the maximum eight-neighborhood sub-maximum (MENS) can be used to filter out background interference. In IMR-PF, a set of MENS local intensity-weighted gradient filters is employed to extract target area features.

**Figure 2:**
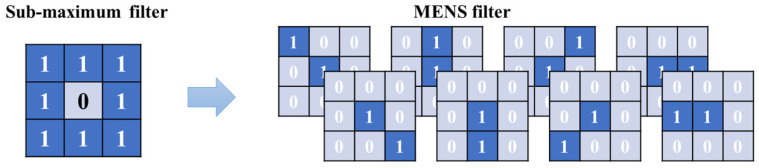
MENS filter diagram.

The MENS filter contains eight sub-filters, each of which corresponds to a neighborhood in a direction. By applying each sub-filter to the original image through convolution and calculating the global maximum, the filter template $f_{SF}^{l}$ is shown in Figure 2, which is a ring structure with a size of 3 × 3. By convolving the original image $I_{O}$, we can easily obtain the sub-maximum filter map $m_{B_{l}}$. The process can be summarized as follows:

(8) $$m_{B_{l}}=I_{O}⊗f_{SF}^{l}$$

In order to more accurately characterize the continuity of spatial distribution between frames, we propose a method to improve image entropy based on multi-scale regional measure. The improved image entropy calculation method is as follows:

(9) $$\begin{array}{l} G_{j}=Downsample(G_{j-1}),G_{0}=I_{0}\\ m_{Gbl}^{j}=G_{j}⊗f_{SF}^{l}\\ m_{B_{l}}^{k}=fuse\_or\_upsample\left(m_{Gbl}^{0},m_{Gbl}^{1},\ldots ,m_{Gbl}^{j}\right)\\ d_{EB}\left(B_{l}^{k},B_{l}^{k-1}\right)=\nabla \left(m_{B_{l}}^{k},m_{B_{l}}^{k-1}\right),l=1,\ldots ,8\\ dm_{Bl}=d_{EB}\left(B_{l}^{k},B_{l}^{k-1}\right)\sum_{h=1}^{m}p_{I_{h}}\log_{2}p_{I_{h}} \end{array}$$

Among them, $m_{B_{l}}^{k}$ and $m_{B_{l}}^{k-1}$ are the sub-maximum filter feature maps of the corresponding areas of two adjacent frames of images, m is the number of pixels with different grayscale values in the window, and $p_{I_{h}}$ is the probability of the occurrence of a pixel value with grayscale $I_{h}$.

Finally, the improved image entropy is normalized to obtain the multi-scale target area measure, and the sum of the improved image entropy reflecting the target mutation state is denoted by $dm_{Bl}^{*}$.

(10) $$S_{Em}=\sum_{l}^{8}dm_{Bl},dm_{Bl}^{*}=\frac{dm_{Bl}}{S_{Em}}$$

#### 3.3.2. Improved Firefly Algorithm Based on Multi-Scale Area Measure (IMFA)

The resampling process of the PF involves duplicating high-weighted particles while discarding low-weighted particles. Nevertheless, this can lead to a particle impoverishment issue in severely degraded particle sets. The Firefly algorithm, employing swarm intelligence, was introduced as a solution to this problem. In the standard firefly algorithm, particles move toward high-brightness particles with a fixed step factor. The literature [11] has verified the correlation between the PF algorithm enhanced by the Firefly algorithm and the particle likelihood. When the tracking state changes suddenly, particle optimization guided by a priori tends to increase the inertial error.

Furthermore, particle interactions significantly increase computational complexity. Therefore, an enhanced firefly algorithm is proposed to address weight degradation and particle scarcity issues through improved resampling and particle distribution.

At the same time, by incorporating target motion characteristics, the motion state reflected by the image measure is integrated to guide particle optimization. This enables dynamic adjustment of the particle group’s motion trend, ensuring that most particles gravitate towards high-likelihood regions during sudden motion changes, thereby augmenting the algorithm’s ability to handle unexpected motion scenarios. We define the relative intensity and relative distance of particles based on the latest measure. The particle with the highest intensity determines the direction of movement of other particles, while the attraction determines the distance of movement of particles. In the improved firefly algorithm, the maximum attraction is 0.85 and the light absorption coefficient is 1.

(11) $$I_{new}^{i}\left(k\right)=I_{0}\times e^{-\eta \left|z_{new}-z_{pred}^{i}\right|}$$

Among them, $z_{new}$ is the latest measure value and $z_{pred}^{i}$ is the predicted observation value of particle i.

The attraction of particle i is improved by using multi-scale regional measure:

(12) $$\beta_{dm}^{i}(k)=dm_{Bl}^{*}\times \left(1-\beta_{0}\times e^{-\gamma r_{igbest}^{2}}\right)$$

where $z_{igbest}$ is the distance between particle i and the optimal estimated particle $z_{gbest}$. In the improved method, the more drastic the change in the target area, the stronger the attraction, which makes the particle closer to the likelihood area.

In the particle position update, a fixed update step factor may cause the firefly to vibrate near the optimal value, resulting in a decrease in calculation accuracy. Therefore, we use a variable update step in the particle position update equation,

(13) $$\begin{array}{l} x_{k,dm}^{i}=\;x_{k}^{i}+\beta_{k,dm}^{i}\times \left(x_{k}^{gbest}-x_{k}^{i}\right)+d_{k}^{i}\times \alpha \times \left(rand-\frac{1}{2}\right)\\ d_{k}^{i}=\frac{\left|x_{k}^{gbest}-x_{k}^{i}\right|}{d_{\max}}\\ d_{\max}=\max\left\{\left|x_{k}^{gbest}-x_{k}^{i}\right|,i=1,2,\ldots ,N\right\} \end{array}$$

where α is a step factor parameter, and rand refers to a random factor [0, 1] that obeys a uniform distribution and is used to represent a random perturbation in the firefly algorithm. $d_{\max}$ is the maximum value of the distance between the best particle $z_{gbest}$ and other particles.

#### 3.3.3. Improved Firefly Algorithm Optimized Particle Filter Based on Multi-Scale Regional Measure (IMR-PF)

The standard PF algorithm suffers from a degeneracy problem, where the weights become concentrated on only a few particles after several iterations. In addition to improving the resampling method, a suitable choice of importance density can alleviate this issue. Although the standard PF utilizes an easily implementable prior distribution $p\left(x_{k}|x_{k-1}\right)$, it lacks the integration of new observation, causing the filter to degenerate quickly. To mitigate these problems, this method employs the EKF to sub-optimally approximate the optimal importance density. The mean and variance of the *i* th particle are calculated using the EKF and the latest observation information $z_{k}$, to approximate the optimal importance density, subsequently utilizing these statistics to sample and update particles.

It is assumed that the state transition equation and measure equation of the system model are:

(14) $$\begin{array}{l} x_{k}=F\left(x_{k-1},u_{k-1}\right)+w_{k-1}\\ z_{k}=H\left(x_{k}\right)+v_{k} \end{array}$$

where $x_{k}$ is the state vector, $z_{k}$ is the measure value at time k, $x_{k-1}$ is the state vector at time k−1, F is the state transfer matrix, and H is the measure matrix. $v_{k}$ and $w_{k-1}$ are the system and measure noise, respectively.

$v_{k}$ and $w_{k}$ are represented by covariance matrices for uncorrelated zero-mean Gaussian noises $R_{k}$ and $Q_{k}$, respectively.

The extended Kalman filter process is,

(15) $$\begin{array}{l} x_{k,pre}^{i}=F(x_{k-1}^{i})\\ P_{k,\rho re}^{\left(i\right)}=F_{k}^{\left(i\right)}P_{k-1}^{\left(i\right)}F_{k}^{T(i)}+Q_{k-1}\\ K_{k}=P_{k,pre}^{\left(i\right)}H_{k}^{T(i)}[C_{k}^{i}R_{k}C_{k}^{T(i)}+R_{k}{]}^{-1}\\ {\bar{x}}_{k}^{\left(i\right)}=x_{k,pre}^{i}+K_{k}(z_{k}-H(x_{k,pre}^{i}))\\ {\hat{P}}_{k}^{\left(i\right)}={\hat{P}}_{k,pre}^{\left(i\right)}-K_{k}H_{k}^{\left(i\right)}P_{k,pre}^{\left(i\right)} \end{array}$$

Using the latest observations $z_{k}$, we calculate the mean and covariance of the estimated *i*th particle in the step k as,

(16) $$\begin{array}{l} {\bar{x}}_{k}^{\left(i\right)}=x_{k,pre}^{i}+K_{k}(z_{k}-H(x_{k,pre}^{i}))\\ {\hat{P}}_{k}^{\left(i\right)}={\hat{P}}_{k,pre}^{\left(i\right)}-K_{k}H_{k}^{\left(i\right)}P_{k,pre}^{\left(i\right)} \end{array}$$

In the context of the improved particle filter algorithm, the recommended density distribution of particle *i* generated by the extended Kalman filter algorithm can be obtained, and then the firefly algorithm improved based on image measure, which is used for resampling:

(17) $$x_{k}^{i}\sim q(x_{k}^{i}|x_{0:k-1}^{i},z_{1:k})=N(x_{k}^{i},{\hat{P}}_{k}^{\left(i\right)})$$

After obtaining the particle estimation value $x_{k,dm}^{i}$ and observation target $z_{k}$ of the frame k, the particle weight $\omega_{k}^{i}$ can be calculated by the transition probability density function $p\left({\bar{x}}_{k}^{i}|{\bar{x}}_{k-1}^{i}\right)$, the importance function $q\left({\bar{x}}_{k}^{i}|{\bar{x}}_{k-1}^{i},y_{1:k}\right)$ and the likelihood probability density function $p\left(y_{k}|{\bar{x}}_{k}^{i}\right)$. For the multivariate normal distribution, the importance weight of the particle can be approximately replaced by the likelihood probability density, and the particle weight $\omega_{k}^{i}$ is given by,

(18) $$\begin{array}{l} \omega_{k}^{i}=\omega_{k-1}^{i}\frac{p\left(y_{k}|{\bar{x}}_{k}^{i}\right)p\left({\bar{x}}_{k}^{i}|{\bar{x}}_{k-1}^{i}\right)}{q\left({\bar{x}}_{k}^{i}|{\bar{x}}_{k-1}^{i},y_{1:k}\right)}\\ \propto \frac{1}{(2\pi )|R_{k}|1/2}\exp\left(\frac{-{\left[y_{k}-H\left({\hat{x}}_{k}^{i}\right)\right]}^{T}R_{k}^{-1}\left[y_{k}-H\left({\hat{x}}_{k}^{i}\right)\right]}{2}\right) \end{array}$$

The particle weights are normalized to more accurately approximate the state posterior probability density function. The normalized weight is,

(19) $${\tilde{\omega }}_{k}^{i}=\frac{\omega_{k}^{i}}{\sum_{i=1}^{N}\omega_{k}^{i}}$$

Therefore, the state of the target to be tracked is the weighted average of the particles, calculated as,

(20) $${\hat{x}}_{k}=\sum_{i=1}^{N}{\tilde{\omega }}_{k}^{i}x_{k,dm}^{i}.$$

## 4. Experiments

### 4.1. Experimental Setup

All algorithms were implemented on a PC equipped with a GTX 1660Ti GPU and an Intel (R) Core (TM) i7-10700 CPU @ 2.9 GHz, running on a Windows 10 operating system with the pytorch1.90 software environment. The GTX 1660Ti GPU is produced by NVIDIA in Santa Clara, CA, USA. The Intel (R) Core (TM) i7-10700 CPU is produced by Intel in Santa Clara, CA, USA. The experimental system includes a two-dimensional rotating platform integrated with a high-resolution wide-field camera and a close-up camera. The wide-field camera used is the MS-XG903C/M, which captures images at a resolution of 4208 × 2160 and a frame rate of 100 fps. The images acquired by this camera serve as the primary information source for the system, with full-resolution images being processed by the proposed adaptive digital zoom algorithm and the IMR-PF algorithm. The close-up camera is utilized solely for the auxiliary verification of experimental results and detailed observation of targets, ensuring accurate validation of the tracking performance. When the hardware system was equipped with algorithms for detection and tracking, we used the pixel difference between the target position and the center of image as the miss amount, which was used as the error of the PID control of the two-dimensional rotating platform. The two-dimensional rotating platform was driven to rotate in real time, while the large-field-of-view camera and the close-up camera also followed the target with the system. For the detection of long-distance fast-moving targets, we utilized the advanced deep learning-based Yolov8 [41] target detection algorithm as the target detection base-method of our system, which was optimized for GTX1660Ti through Tensor-RT. The confidence threshold was set according to the official recommendation at conf = 0.3.

In the tracking experiments, our method was compared with other state-of-the-art methods. The IMR-PF algorithm is associated with four improved methods based on KCF-PF [37], IFA-PF [11], FAPF [42], and PSO-PF [35] to evaluate the performance of the IMR-PF. Numerous deep learning models have been applied to object tracking, and we have conducted comprehensive comparisons with several state-of-the-art methods. These include Siamese network trackers based on deep feature matching (Siam RPN++) [12], multi-local guided trackers (MLGT) [33], tracking models utilizing adaptive update strategies with multiple attention mechanisms and background features (FBST) [31], and Siamese object tracking models integrated with re-identification modules (SPT) [30]. Additionally, we demonstrated the superiority of IMR-PF over other tracking techniques by comparing it with SORT based on Kalman filter [26], ByteTrack [27], oc-sort using momentum consistency and inverse Kalman filter [43], and EKF [25].

To evaluate the effect of the adaptive ROI long-range object detection and tracking algorithm, we also applied it to detect and track objects with significant scale changes.

### 4.2. Dataset

In the method validation experiments, we selected both public datasets and partially self-made datasets as testing sources. Among these, the VOT2021 dataset [44] includes 51 target tracking test sets, incorporating factors such as occlusion, camera movement, lighting changes, size variations, and target motion. Specifically, VOT2021 contains: over 30% of data with rapid motion (defined as target displacement exceeding 50% of the target size between frames); over 25% of data with small targets (defined as targets occupying 1% to 5% of the image area); and over 40% of data with target changes (defined as scale variations exceeding 2 times). To comprehensively quantify the robustness of our method to challenges such as target changes and rapid motion, we selected video segments from the VOT2021 dataset that exhibit significant size variations and include five interfering factors. The details of the selected video segments are provided in Table 1.

Additionally, to evaluate the effectiveness of our method and system in practical applications, we created a self-made dataset consisting of real videos capturing long-distance, fast-moving targets in U-shaped skiing competitions. This self-built dataset has a resolution of 4096 × 2160 and includes 5 video clips, each containing 340 frames. The target size in this dataset ranges from 30 × 30 to 510 × 510 pixels, and all the data feature the characteristics of target motion and target deformation.

By combining these datasets, we ensured a thorough evaluation of our method’s performance across diverse and challenging scenarios.

### 4.3. Evaluation Criteria

To quantitatively evaluate different methods, we used the central error (CE), overlap rate (OR), the value of AUC (area under the curve), and the success rate plot (SP) as evaluation metrics.

The CE is defined as,

(21) $$CE(k)=\sqrt{{\left(x_{T}^{k}-x_{G}^{k}\right)}^{2}-{\left(y_{T}^{k}-y_{G}^{k}\right)}^{2}}$$

where $\left(x_{G}^{k},y_{G}^{k}\right)$ represents the true center position of the target and $\left(x_{T}^{k},y_{T}^{k}\right)$ represents the center position obtained by the tracker. A good tracker should accurately track the target position in real time, so the CE value of the best tracker is expected to be small.

The OR evaluates the quality of the tracker by the ratio of the intersection and union of the bounding box obtained by the tracker and the bounding box of the true position,

(22) $$OR(k)=\frac{area(R_{T}^{k}\cap R_{G}^{k})}{area(R_{T}^{k}\cup R_{G}^{k})}$$

where $R_{G}^{k}$ represents the bounding box of the ground truth and $R_{T}^{k}$ represents the bounding box of the tracker in the *k*th frame.

The SP represents the success rate curve corresponding to the correct tracking threshold for different overlap rates, and the value of AUC is the area value under the curve in the success rate graph. Precision reflects the performance of a tracking algorithm by measuring the distance between the estimated target position and the center of the ground truth at various thresholds.

### 4.4. Target Tracking Experiment on U-Skier Dataset

Our method aims to solve the problem of poor tracking performance due to changes in distance and target. Initially, we first conducted a comparative test on the self-built U-skier dataset. In the simulation experiment, we used the full-size U-skier video as the input source, detected and tracked the target through different tracking algorithms and the proposed algorithm, and compared and evaluated the tracking results of the algorithms.

**Figure 3:**
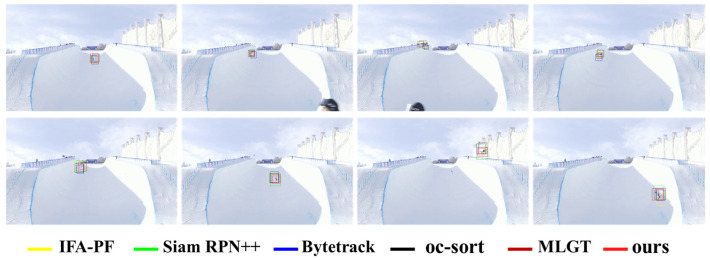
Tracking results of the top six methods on the U-skier dataset (different video clips from top to bottom).

Qualitative experiment: Figure 3 illustrates the tracking results of each tracker when the target changes dramatically. For the sake of clarity, we only show the results of the six methods with the best performance in Figure 3. As can be seen from the figure, the tracking results of the IFA-PF are better than the comparison method, and the target box is most consistent with the real target box. Secondly, ByteTrack also has a good effect. It can enhance the precision of target tracking by associating low-score detection boxes. However, the IFA-PF method predicts a larger target box, which may be because the particle filter in IFA-PF averages the filtering results, making the tracking box change smoother. The Siam RPN++ method obtains relatively poor results, which may be due to the image blur caused by target changes, which affects the discriminability of the model. Compared to the Siam RPN++ method, the MLGT approach exhibits smaller positional deviations in its prediction results. This improvement can likely be attributed to the incorporation of contextual information through self-attention and cross-attention mechanisms, which enhance the precision of target localization. However, when compared to the method proposed in this study, the MLGT’s prediction bounding boxes are notably larger, suggesting that our approach achieves more precise target delineation while maintaining superior localization accuracy.

Quantitative experiment: For the tracking results of each method, Table 2 describes the average center error and the time required for tracking calculation. It can be seen that our method achieves the smallest error and a high success rate.

**Figure 4:**
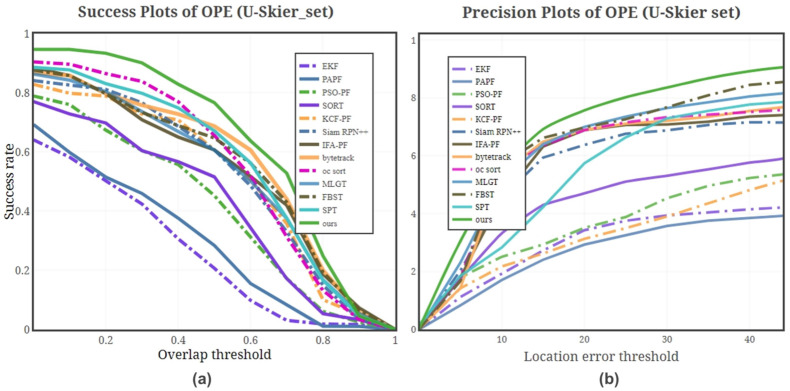
Evaluation result on the U-skin dataset. (**a**), Success rate of different methods on the U-skier dataset; (**b**), Precision of different methods on the U-skier dataset.

Figure 4 illustrates the success rate and precision of different methods. It is evident that KCF-PF and PSO-PF exhibit better results compared to the FAPF algorithm, indicating that the use of KCF and swarm intelligence optimization can improve the effect of PF. Similarly, The Siam RPN++ method performs better results when the overlap rate threshold is small, indicating that the matching of similar image features can significantly improve the effect of the algorithm. However, as the overlap rate threshold increases, Siam RPN++ suffers from a decline in the feature matching effect, and its advantages diminish. Algorithms including Kalman filtering follow a similar trend. The Bytetrack and oc-sort methods demonstrate better robustness to state mutations of the target when the overlap rate threshold is low. The incorporation of contextual features allows MLGT to achieve a higher success rate at low overlap thresholds. The tracking success rate, measured by Intersection over Union (IoU) with a confidence interval of 0–50%, demonstrates that the proposed method improves the success rate by at least 5% compared to other leading methods. Deep learning-based methods generally exhibit higher success rates than non-deep learning methods, except for the proposed method. However, when the confidence interval exceeds 50%, deep learning-based methods, such as MLGT and FBST, show lower success rates compared to traditional methods. Although the success rate of the proposed method also decreases in this range, it still outperforms other methods, further proving that resolution is a critical factor influencing tracking success rates.

However, the precision curve reveals that this method underperforms at low position error thresholds, which restricts its applicability in large-scale scenarios where precise localization is critical. Our method combines Kalman filtering and particle filtering with image measure features to achieve better results regardless of the size of the threshold. The tracking error table demonstrates that our method is shown to improve temporal resolution through time-consuming comparison results, which has significant advantages in the case of high-resolution images. It can be observed that while traditional tracking methods such as KCF-PF and PSO-PF, along with recent deep learning-based approaches, demonstrate advantages in terms of success rates, their precision curves reveal relatively poor performance. This suggests that these methods are capable of accurately estimating the general region of the target’s features but struggle to pinpoint the exact location, likely due to limitations in resolution. Such a trade-off highlights the challenges these methods face in achieving both robust target localization and high positional accuracy, particularly in scenarios where fine-grained precision is critical.

Real-time Experiment: under identical hardware configurations and experimental conditions, a comparison of computational time consumption on Table 2 reveals that our method significantly improves time resolution, particularly demonstrating notable advantages when processing high-resolution images. Experiments indicate that non-deep learning methods generally incur lower computational costs, making them more suitable for real-time applications. In contrast, the computational cost of MLGT is higher than that of convolutional Siamese networks, primarily due to the extensive time required for cross-attention mechanisms to extract contextual information. Furthermore, compared to the earlier FAPF method, the improved IFA-PF approach shows a substantial reduction in computational consumption. This improvement underscores the effectiveness of optimizing resampling processes in enhancing algorithmic efficiency. By incorporating image measures to further refine resampling, we achieve additional efficiency gains, making our method both robust and computationally efficient for real-time tracking tasks.

### 4.5. Indoor Simulation Experiment on U-Skier Dataset

**Figure 5:**
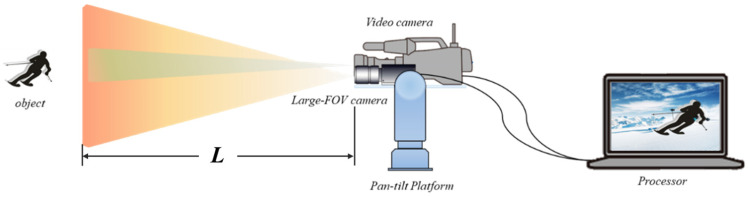
System schematic: large FOV camera is used to capture object images, PC processor is used to target detection and tracking on the captured images, and the tracking results are used to drive the Pan-tilt Platform to rotate. Video camera is a zoom camera used to capture close-up images of the target and evaluate the results. L is the depth range of the entire system.

To verify the actual effectiveness of the detection and tracking system, we conducted a scene simulation experiment, as illustrated in Figure 5. The experiment utilized a 65-inch 4K resolution display to present the U-skier dataset video, simulating a skiing scene with a depth range L of 0–170 m. To further observe and analyze the target captured by the detection and tracking system, we employed a variable focal length video camera (with a focal length ranging from 25 mm to 375 mm) to zoom in and closely monitor the target, enabling more accurate determination of tracking errors.

Due to the close-up camera’s longer focal length compared to the wide-field camera, the simulated scene was set up with a minimum working distance of 4.5 m to ensure clear imaging. The display size for the simulation was 1440 mm × 810 mm, while the CMOS size of the wide-field camera was 8.8 mm × 6.6 mm. Based on the proportional scaling model described in Equation (1), the detected skier’s target size ranged from 18 × 18 to 301 × 301 pixels. During the detection and tracking process, we dynamically adjusted the focal length of the close-up camera based on the target’s pixel size to facilitate detailed observation of the target’s state.

**Figure 6:**
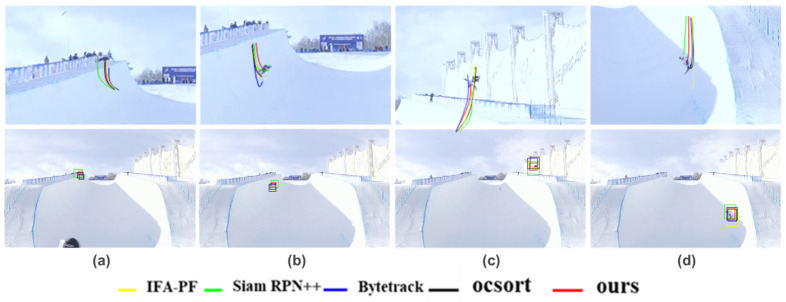
Tracking results of the top five tracking methods on the U-skier dataset in an indoor simulation environment (The following line shows the tracking results on a high-resolution image with a large field of view. The top line shows the close-up camera captured images corresponding to each tracking moment. The trajectory curve in the figure was formed by manually registering the front and rear frames and connecting the predicted center positions of different methods. The images in column (**a**) depict a scenario where the athlete’s target leaps in the distance, with interference from similar targets in the background. The images in column (**b**) show the athlete’s target descending and encountering a sudden change in trajectory due to the ski slope. The images in column (**c**) illustrate the athlete’s target leaping into the air from a close distance. The images in column (**d**) capture the athlete’s target descending at a close distance, experiencing a sudden change in trajectory upon encountering the ski slope).

For the actual tracking result processing, we manually aligned the close-up images of adjacent frames to ensure accuracy and consistency in the analysis. Given the real-time requirements of the tracking system, we focused on presenting the target trajectory results obtained by five different methods, all of which exhibit high real-time performance. These results are illustrated in Figure 6, providing a clear comparison of the tracking precision and robustness of each method under realistic conditions. This approach allows us to evaluate the effectiveness of the proposed system while maintaining the practical constraints of real-time operation.

Qualitative experiment: in order to compare the results intuitively, we show the trajectory tracking results of each tracker when the real motion trajectory of the target changes on the U-skier dataset in Figure 6. Figure 6a,c show that our method can accurately track the target when there are interferences from other human targets and changes in the target’s motion, but SiamRPN++ has tracking errors due to changes in target features. When the target’s motion changes dramatically, algorithms containing the Kalman filter experience position drift issues, as depicted in Figure 6c. It can also be seen from the picture that our method’s target box is most consistent with the real target under significant target changes. Then, the IFA-PF method exhibits a smoother over-prediction problem. This may be because IFA-PF is greatly affected by historical results, leading to failure in predicting the target trajectory when changes occur. The oc-sort method achieves relatively good results, although there remains a significant error in predicting the target box.

**Figure 7:**
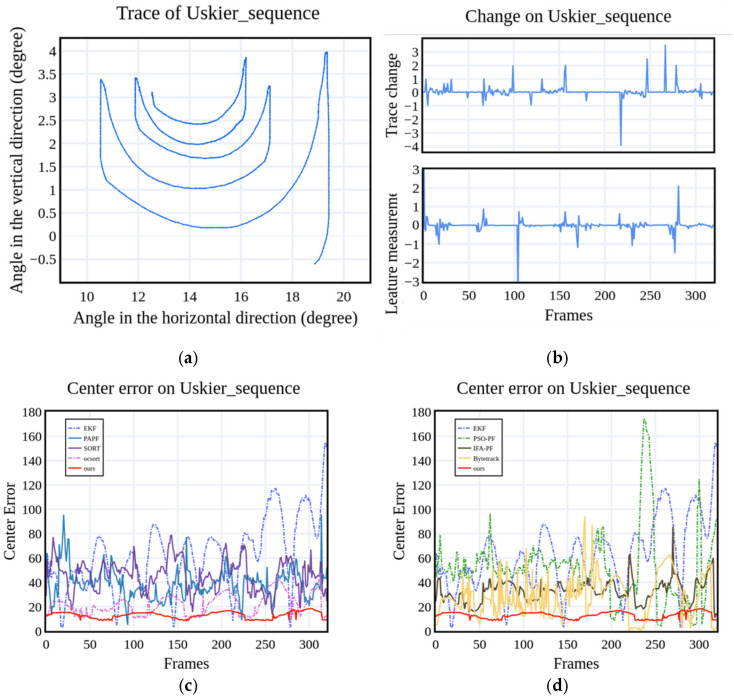
Tracking results on the U-skier dataset in an indoor simulation environment. (**a**) Ground truth of the target trajectory; (**b**) Changes in the target trajectory and target area measure; (**c**,**d**) Tracking error curves of different methods as the video frame changes.

Quantitative experiment: Figure 7 illustrates the target measure and frame-by-frame tracking errors of various methods during target tracking. When the target measure value shows a peak mutation, the errors of each method increase accordingly. Figure 7 shows that the EKF has the largest error, failing to promptly reduce it following an increase in target error. This failure may be attributed to tracking failure. Although the PSO-PF method enhances the tracking success rate compared with the FAPF method, it experiences substantial error increments upon target changes, likely due to the ant colony algorithm being attracted to local optimal values during iterations, limiting the global search ability. The IFA-PF algorithm demonstrates relatively low error, indicating that taking the optimal particle as the center can improve the global search ability after the tracking state mutation. The center error of oc-sort is further reduced. Reference [43] also verifies that alternating reverse checking the parameters of the Kalman filter can reduce the center error of tracking. Our method has the lowest target center error in indoor simulation experiments. Figure 7b shows that the image measure is associated with the target trajectory change state and reflects the target area change. This observation shows that our method is less affected by the change in the target motion state, demonstrating that introducing measure features helps to improve the performance of target tracking.

### 4.6. Experiments on the VOT2021 Dataset

To evaluate the performance and robustness of the proposed method, we conducted experiments on the VOT2021 dataset, a general target tracking dataset. By testing the datasets with six interference factors such as Motion Change, Camera Motion, Occlusion, Background clutter, Size Change, and Illumination Change at the same time, we compared and evaluated this method with other tracking methods.

**Figure 8:**
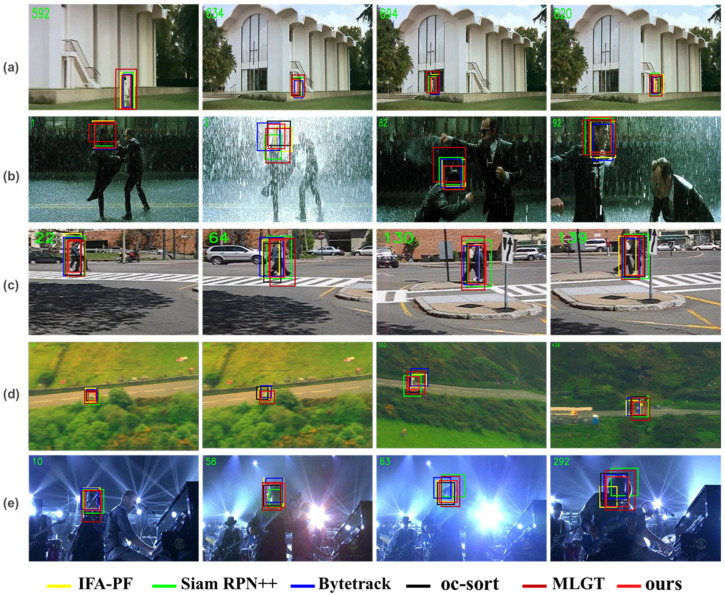
Results of the top six different methods on the VOT2021 dataset for tracking different data segments on the screen. (**a**) represents the success rate of these methods on the graduate_set of the VOT2021; (**b**) represents the success rate of these methods on the matrix_set of the VOT2021; (**c**) represents the success rate of these methods on the pedestrian_set of the VOT2021; (**d**) represents the success rate of these methods on the road_set of the VOT2021; and (**e**) represents the success rate of these methods on the shaking_set of the VOT2021.

Qualitative experiments: in the case of long-term tracking under severe occlusion, our method demonstrated excellent performance. Compared with Kalman filter tracking matching Bytetrack and oc-sort, the image feature discriminant tracker SiamRPN++ can address the problem of tracking failure caused by long-term target loss. As illustrated in Figure 8b,e, when the target changes significantly, the image feature discriminant tracker performs poorly, and the method based on the latest observation center and trajectory matching is more robust. When other targets and background interference are present in the scene, the MLGT method exhibits smaller positional deviations, demonstrating that its use of contextual information effectively mitigates the impact of environmental changes. However, its performance diminishes when tracking small targets, as highlighted by the results from the U-skier dataset. This limitation underscores the influence of resolution as a constraining factor for deep learning-based methods. In contrast, our method enhances algorithmic stability by integrating contextual information into the calculation of target domain image measures. This approach not only improves robustness in complex environments but also addresses the challenges posed by small targets, making it more versatile and effective across a wider range of tracking scenarios.

**Figure 9:**
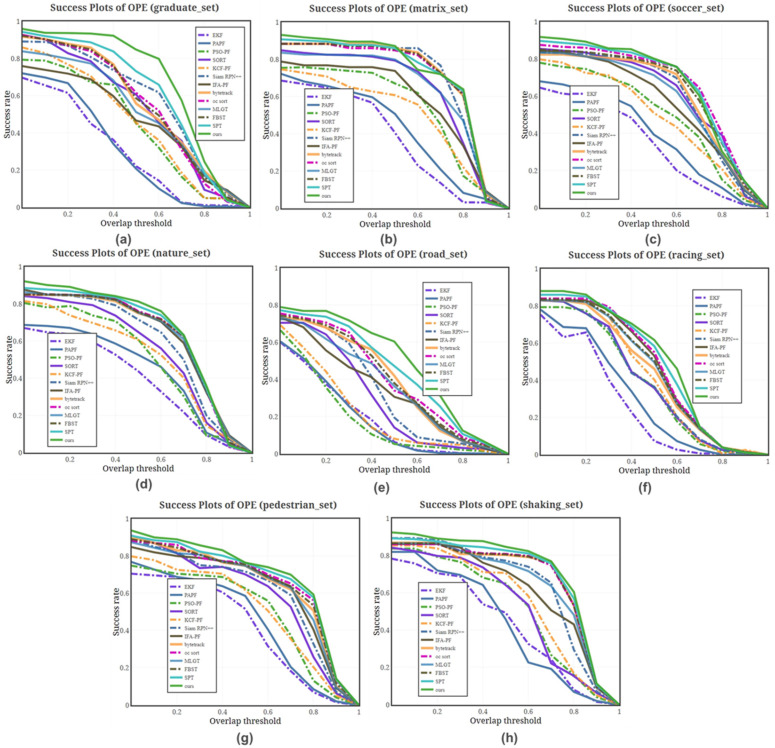
Success rate of different methods on the different sub-datasets of the VOT2021 dataset. (**a**) represents the Success rate of these methods at graduate_set in the VOT2021 dataset; (**b**) represents the Success rate of these methods at matrix_set in the VOT2021 dataset; (**c**) represents the Success rate of these methods at soccer_set in the VOT2021 dataset; (**d**) represents the Success rate of these methods at nature_set in the VOT2021 dataset; (**e**) represents the Success rate of these methods at road_set in the VOT2021 dataset; (**f**) represents the Success rate of these methods at racing_set in the VOT2021 dataset; (**g**) represents the Success rate of these methods at pedestrian_set in the VOT2021 dataset; and (**h**) represents the Success rate of these methods at shaking_set in the VOT2021 dataset.

Quantitative Experiments: Figure 9 further illustrates that the tracking success rate of methods relying solely on the target’s motion state is significantly lower in scenarios where the target is lost for an extended period. This limitation highlights the challenges of maintaining robust tracking when relying exclusively on motion-based information, particularly in cases of prolonged target occlusion or disappearance. This further confirms that appearance similarity is useful for long distances. In Figure 8c, it can be observed that the various methods have very similar results when the target moves smoothly and the changes are not obvious. However, when motion blur and fast motion are present, as shown in Figure 9c,h, many trackers fail due to the limited tracking range, especially when sudden changes occur in the motion trajectory. Consequently, our method enhances the particle filter method by introducing a regional change feature measure. The interference of objects in the datasets corresponding to Figure 9b,e affects the tracking effect. The particle filter method reduces the influence of the interference target by sampling a large number of particles. Figure 6 and Figure 8 show that our method has good performance for both large and small targets.

Overall, when the confidence interval of Intersection over Union (IoU) is between 0 and 50%, as shown in Figure 9a,e and Table 3, our method demonstrates a significant advantage in success rate for datasets with a high proportion of target changes and small target movements. When the confidence interval exceeds 50%, although our method’s success rate converges toward that of other advanced methods as the error range decreases, it still maintains an advantage on datasets with a higher proportion of small targets. This further indicates that our method is robust across different confidence intervals and performs well in diverse and challenging tracking scenarios.

**Figure 10:**
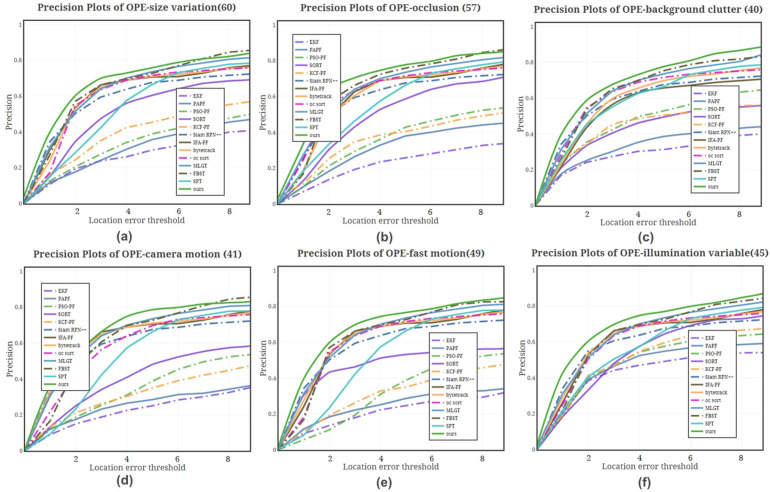
Precision of different methods on data with different properties in the VOT2021 dataset. (**a**) represents the precision of these methods at size variations properties; (**b**) represents the precision of these methods at occlusion properties; (**c**) represents the precision of these methods at background clutter properties; (**d**) represents the precision of these methods at camera movement properties; (**e**) represents the precision of these methods at target fast motion properties; and (**f**) represents the precision of these methods at illumination variable properties.

By analyzing the precision plot for different interference factors in Figure 10, it is evident that lighting changes have a minimal impact on the tracking methods. In contrast, EKF and FAPA exhibit relatively large center position prediction errors across various interference factors, likely due to their overly simplistic models. When occlusion occurs, improved particle filtering methods and those incorporating Kalman filtering mechanisms achieve higher accuracy. This improvement can be attributed to the trajectory continuity discrimination, which reduces the impact of occlusion on target detection and tracking. Figure 10c further demonstrates that deep learning models enhanced with contextual information exhibit greater stability under background clutter interference. However, in scenarios involving target motion and size variations, our method achieves the highest accuracy. When combined with the accuracy results for other interference factors, our approach consistently outperforms others, underscoring its robustness and adaptability in diverse and challenging tracking environments. This comprehensive performance highlights the effectiveness of our method in maintaining precision and stability across a wide range of conditions.

## 5. Ablation Study

To verify the effectiveness of the different components designed in our method, we conducted ablation experiments on the U-skier dataset with the same settings as Section 4.1. Specifically, we compared the two improved submodules:

(1) Adaptive digital zoom structure: this structure is capable of handling both small targets at a distance and large targets at a close distance by adaptively cropping and scaling the visual sensor image. It ensures a uniform target size in the input detection network. High-resolution and large-field-of-view images are time-consuming for target detection. This structure can reduce unnecessary interference while reducing the amount of algorithm calculation, so that the detection and tracking algorithms can focus on effective target features.
(2) Multi-scale feature measure of target region: this model enhances the resampling rule of particle update and exhibits high responsiveness to significant target changes. By introducing the mutation type of target motion and target change information into the tracking algorithm, this structure facilitates improving tracking performance.

To validate the effectiveness of the two submodules, we conducted a comparative analysis of the improved method, evaluating the impact of including each submodule separately and both submodules simultaneously. Additionally, to assess the influence of the object detection base model on the enhanced tracking module, we selected YOLOv8 [41] and YOLOv5 [45] as base models for comparison, as both are widely recognized for their leading performance in object detection tasks. For the object detection model, we chose the official recommended confidence level, conf = 0.3.

**Figure 11:**
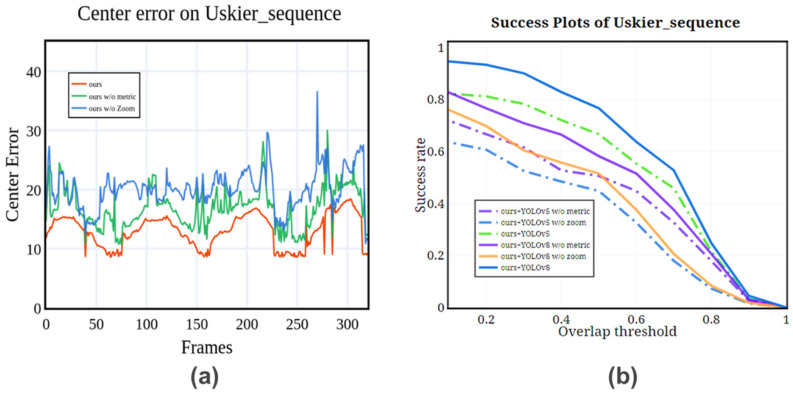
Tracking error and success rate curves of different module improvement methods on the U-skier dataset. (**a**) Representing the central error of methods with different structures at Uskier sequence; (**b**) Repeat the success rate of methods with different structures at Uskier sequence.

As illustrated in Figure 11, the adaptive digital zoom structure, primarily designed to enhance the real-time performance of high-resolution and large field-of-view detection, also contributes to improved target tracking accuracy. Furthermore, the target area feature measurement module significantly enhances the overall performance of the algorithm, confirming the necessity of our proposed model improvements. By comparing different base models, we observed that our proposed modules consistently improve tracking accuracy across various detection models, demonstrating the broad applicability of our method.

From the central error curve, it is evident that the introduction of the adaptive zoom structure uniformly reduces target tracking errors. Moreover, incorporating measurement features effectively mitigates errors caused by target mutations without significantly increasing computational time. These results underscore the robustness and efficiency of our approach in achieving high-precision tracking while maintaining real-time performance.

## 6. Conclusions

In general, we present a novel improved particle filter algorithm named IMR-PF for visual tracking. The diverse and accurate distribution of particles is destroyed in the standard resampling process of Particle filter. To address this issue, we combine PF with regional image measure and KF, resulting in an enhanced overall accuracy of the particle filter algorithm and improved performance when dealing with target mutations. In addition, we utilize an adaptive zoom algorithm to deal with the problems of long distance and target size variation, which reduces the interference of the PF tracking algorithm and allows particles to be more biased towards high-likelihood areas. The proposed IMR-PF is validated on the VOT2021 dataset and our custom U-skier dataset, demonstrating its robustness in various thought-provoking situations. Simulation results show that the ROI adaptive digital zoom tracking system has remarkable tracking performance. We also observed that our method has a more significant impact on tracking success rates compared to deep learning models, particularly at high overlap thresholds. To address the issue of limited accuracy at these thresholds, we plan to integrate contextual deep feature extraction with the current object tracking module in future work. At the same time, we also plan to draw on the advantages of combining shallow features with deep information to solve target tracking problems in more complex environments.

Furthermore, we will explore the multi-target tracking problem based on the proposed IMR-PF framework and investigate its applicability in multi-viewpoint object tracking scenarios. This expansion will not only broaden the scope of our method but also provide valuable insights into addressing more challenging real-world tracking tasks.

## Figures and Tables

**Table 1:** The tracking example of VOT2021 attributes.

Dataset	Frame Numbers	Challenging Factors
Graduate	844	Low Resolution, Motion Change, Camera Motion, Illumination Change, Size Change, Occlusion
Matrix	100	Occlusion, Camera Motion, Illumination Change, Motion Change, Scale Variation
Nature	999	Size Change, Camera Motion, Occlusion, Illumination Change, Motion Change,
Racing	156	Key Frame, Camera Motion, Illumination Change, Motion Change, Occlusion, Size Change
Road	558	Out-of-Plane Rotation, Background Clutters, Deformation, Fast Motion, Size Change, Illumination Change, Occlusion, Camera Motion
Shaking	365	Motion Change, Camera Motion, Occlusion, Size Change, Illumination Change
Soccer1	392	Camera Motion, Illumination Change, Motion Change, Occlusion, Scale Variation
Pedestrian1	140	Size Change, Camera Motion, Illumination Change, Motion Change, Occlusion

**Table 2:** Average center error (ACE) with U-skin dataset and time cost for each method.

	EKF	FAPF	PSO-PF	SORT	KCF-PF	Siam RPN++	IFA-PF	Bytetrack	oc-sort	SPT	FBST	MLGT	Ours
ACE	53.04	39.25	35.37	22.42	41.72	17.40	16.48	24.98	19.46	14.94	15.61	13.53	**12.93**
Time cost (ms)	15.18	43.67	21.38	27.13	12.72	36.31	10.42	16.83	17.16	55.65	43.74	82.64	**8.40**

**Table 3:** Average center error (ACE) with VOT dataset.

	EKF	PAPF	PSO-PF	SORT	KCF-PF	Siam RPN++	IFA-PF	Bytetrack	oc-sort	SPT	FBST	MLGT	Ours
graduate	66.47	23.46	9.43	15.78	4.68	6.74	4.01	9.99	2.08	2.41	2.94	2.59	**1.33**
matrix	55.18	43.67	21.38	7.13	12.72	6.3	5.42	5.83	5.16	5.17	5.81	4.82	**4.40**
nature	33.42	21.58	8.12	14.1	4.03	3.36	3.47	2.71	**1.97**	2.47	2.55	2.27	2.07
racing	25.37	15.04	11.02	1.25	2.2	2.89	2.93	1.44	2.77	1.87	1.98	1.8	**1.03**
road	40.81	35.51	17.16	10.25	7.57	17.09	6.98	8.73	8.10	4.29	4.48	3.88	**3.71**
shaking	59.22	44.75	10.49	5.71	4.94	4.64	4.97	8.91	3.99	4.48	4.67	4.22	**2.88**
soccer1	59.07	45.54	11.42	6.35	3.92	4.82	5.27	7.46	**3.66**	4.66	6.93	4.22	4.03
pedestrian	27.99	25.55	16.18	5.77	2.88	3.13	5.02	5.05	3.87	4.27	4.46	4.03	**2.75**

## Data Availability

The data that support the findings of this study are not openly available due to reasons of sensitivity and are available from the author upon reasonable request.
